# Temperature Effects on the Wind Direction Measurement of 2D Solid Thermal Wind Sensors

**DOI:** 10.3390/s151229771

**Published:** 2015-11-30

**Authors:** Bei Chen, Yan-Qing Zhu, Zhenxiang Yi, Ming Qin, Qing-An Huang

**Affiliations:** Key Laboratory of MEMS of the Ministry of Education, Southeast University, Nanjing 210096, China; chenbei813@163.com (B.C.); zyqseu@foxmail.com (Y.-Q.Z.); xp@seu.edu.cn (Z.Y.); mqin@seu.edu.cn (M.Q.)

**Keywords:** thermal wind sensors, wind direction, temperature effect

## Abstract

For a two-dimensional solid silicon thermal wind sensor with symmetrical structure, the wind speed and direction information can be derived from the output voltages in two orthogonal directions, *i.e*., the north-south and east-west. However, the output voltages in these two directions will vary linearly with the ambient temperature. Therefore, in this paper, a temperature model to study the temperature effect on the wind direction measurement has been developed. A theoretical analysis has been presented first, and then Finite Element Method (FEM) simulations have been performed. It is found that due to symmetrical structure of the thermal wind sensor, the temperature effects on the output signals in the north-south and east-west directions are highly similar. As a result, the wind direction measurement of the thermal wind sensor is approximately independent of the ambient temperature. The experimental results fit the theoretical analysis and simulation results very well.

## 1. Introduction

A typical calorimetric two-dimensional silicon thermal wind sensor usually has a symmetrical structure. The sensor consists of a central temperature sensing element, several heaters and some other temperature sensing elements which are distributed symmetrically around the heaters [1,2]. The heaters warm up the chip above the ambient temperature. With the help of the central temperature sensing element and external control circuits, the temperature difference between the sensor chip and the airflow can be a constant (named the Constant Temperature Difference mode) [3,4,5,6,7]. When an airflow passes over the sensor chip, the temperature profile around the heaters will be asymmetric and the temperature sensing elements around the heaters can measure this distorted temperature distribution. Eventually the output signals in two orthogonal directions can be used to determine the wind speed and direction.

According to whether thermal insulation is present or not, calorimetric flow sensors can be divided into two categories. One type is those with thermal insulation, which are usually fabricated on silicon dioxide membranes [6,8,9,10,11,12], thin crystalline silicon membranes [13,14,15], or on porous silicon layers [16,17]. The thermal insulation is so effective that the total power consumption of some thermal flow sensors can be decreased to the milliwatt or even sub-milliwatt range [11,15], which is attractive for some applications, such as biological and medical applications. In these situations, fluids cannot endure a large temperature increase. Though some sensors with thermal insulation could be integrated with the signal-conditioning circuits on a single chip, the fabrication steps to integrate the micromachined-based sensors within the Complementary Metal Oxide Semiconductor (CMOS) process flow must use compatible materials, thermal budget and highly-selective MEMS release techniques [18]. Besides, the suspended membranes are relatively fragile and not compatible with further silicon processing. In addition, the fabrication of diaphragms easily induces imperfections, such as misalignment of the diaphragm and the structure on it and non-uniformity of the thin film thickness, which could result in an initial offset of the output signal in the absence of flow. Due to the fragility of the membranes, these sensors are usually packaged in tubes which limits their two-dimensional applications. The other type is the thermal flow sensors without thermal insulation. These sensors are mostly realized on thick silicon substrates by using a standard CMOS process [1,19,20]. The use of standard CMOS technologies allows the combination of the sensor and signal-conditioning electronics on a single chip. Moreover, due to their simple structures and high reliability, the packaging of the sensors is fairly straightforward. The sensors can be directly glued onto a thin ceramic disk [20]. In this way, the sensor can be protected from the contamination or destructive effects of the external environment, thus making these sensors reliable for harsh applications. Because thermal wind sensors often work outdoors, in this paper we focus on the second type, which can be called “solid thermal wind sensors”.

In our previous works, the effect of the ambient humidity on solid thermal wind sensors has been investigated [21]. Besides, a semi-empirical model has been established to perform temperature compensation during the wind speed measurement of a solid wind sensor [22]. However, to our knowledge, the influence of the ambient temperature on the wind direction has never been explored.

In this paper, a thermal model of the wind direction measurement is presented, which shows that the wind direction is a function of the output voltages in the north-south and east-west directions. Simulations and experiments are performed to study the temperature characteristics of the output signals in these two orthogonal directions. It is found that the thermophysical properties of the airflow have little impact on the output signals, whereas the thermal conductivities of the substrates play an important role. Finally, with the proposed thermal model, the ambient temperature effect on the wind direction measurement of a solid thermal wind sensor is presented.

## 2. Theoretical Analysis 

**Figure 1:**
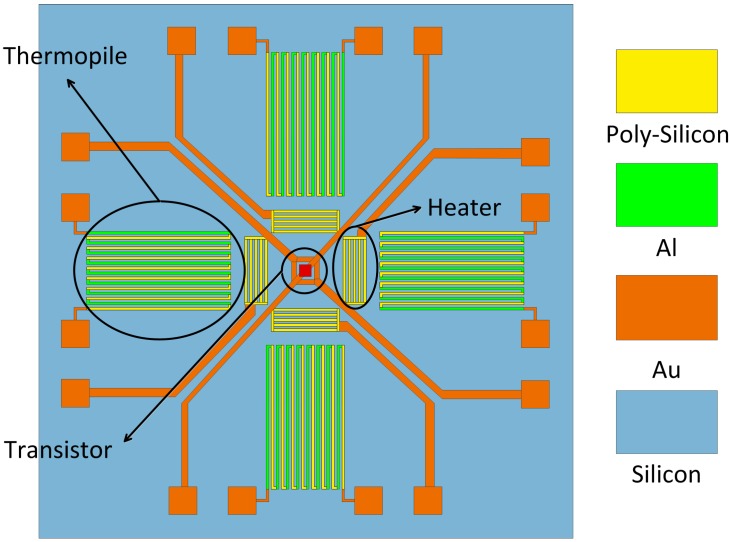
The layout of the thermal wind sensor. Four heaters heat the whole chip. A central transistor measures the average temperature of the sensor chip, while four thermopiles measure the temperature difference between the upstream and downstream sensor surfaces.

As shown in Figure 1, the two-dimensional thermal wind sensor consists of four heating resistors, four thermopiles and a transistor. The central diode (implemented as a diode-connected substrate Positive-Negative-Positive (PNP) transistor) measures the average temperature of the sensor. The four integrated resistors are symmetrically placed around the center point of the sensor and warm up the sensor to above the ambient temperature. The four thermopiles measure the temperature differences between the upstream and downstream sensor surfaces. The sensor works in the constant temperature difference mode (CTD mode). In our previous work [22], the temperature effect on the output signal of the sensor *V_out_* has been presented:

(1) $$V_{out}=\left(A+B\cdot T_{amb}\right)\cdot U^{\frac{1}{2}}$$

It is shown that the output voltage increases linearly with the ambient temperature. Here parameters *A* and *B* are functions of the thermophysical properties of the airflow, the thermal conductivities of the silicon and ceramic substrate, and the Seebeck coefficients of the thermocouples. *T_amb_* is the ambient temperature, and *U* is the wind speed.

**Figure 2:**
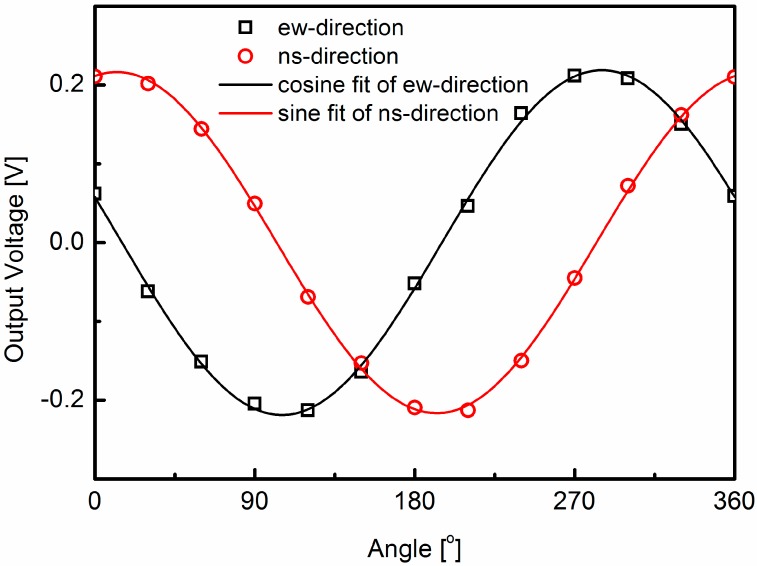
The output voltages in the north-south direction and east-west direction. The wind speed is 7 m/s, and the ambient temperature is 15 °C. According to the fit curves, the constant phase shifts *ε_ns_* and *ε_ew_* are 77.3° and 76.7°, respectively.

The two-dimensional direction sensitivity is obtained by measuring the temperature difference in the two orthogonal directions, the north-south and east-west directions [1]. As shown in Figure 2, the output signals in the north-south and east-west directions can be accurately represented by sine and cosine functions of the wind direction, φ:

(2) $$V_{ns}=\left(A_{ns}+B_{ns}\cdot T_{amb}\right)\cdot U^{\frac{1}{2}}\cdot \sin\left(\phi +\varepsilon_{ns}\right)$$

(3) $$V_{ew}=\left(A_{ew}+B_{ew}\cdot T_{amb}\right)\cdot U^{\frac{1}{2}}\cdot \cos\left(\phi +\varepsilon_{ew}\right)$$

Here, *V_ns_* and *V_ew_* are the output voltages in the north-south direction and the east-west direction, *A_ns_*, *B_ns_*, *A_ew_* and *B_ew_* are the temperature compensation parameters in the two directions, and *ε_ns_* and *ε_ew_* are the constant phase shifts. The constant phase shifts reflect the fact that the sensor’s reference axis is not aligned with the reference axis of the measurement system. According to the fit curves in Figure 2, the constant phase shifts ε_ns_ and ε_ew_ are 77.3° and 76.7°, respectively. By using Equations (2) and (3), the wind direction can be expressed with *V_ns_* and *V_ew_* as follows:

(4) $$\phi =\tan^{-1}\left(\frac{\left(A_{ew}+B_{ew}\cdot T_{amb}\right)\cdot V_{ns}\cdot \cos\varepsilon_{ew}-\left(A_{ns}+B_{ns}\cdot T_{amb}\right)\cdot V_{ew}\cdot \sin\varepsilon_{ns}}{\left(A_{ew}+B_{ew}\cdot T_{amb}\right)\cdot V_{ns}\cdot \sin\varepsilon_{ew}+\left(A_{ns}+B_{ns}\cdot T_{amb}\right)\cdot V_{ew}\cdot \cos\varepsilon_{ns}}\right)$$

## 3. FEM Simulation of the Wind Sensor

**Figure 3:**
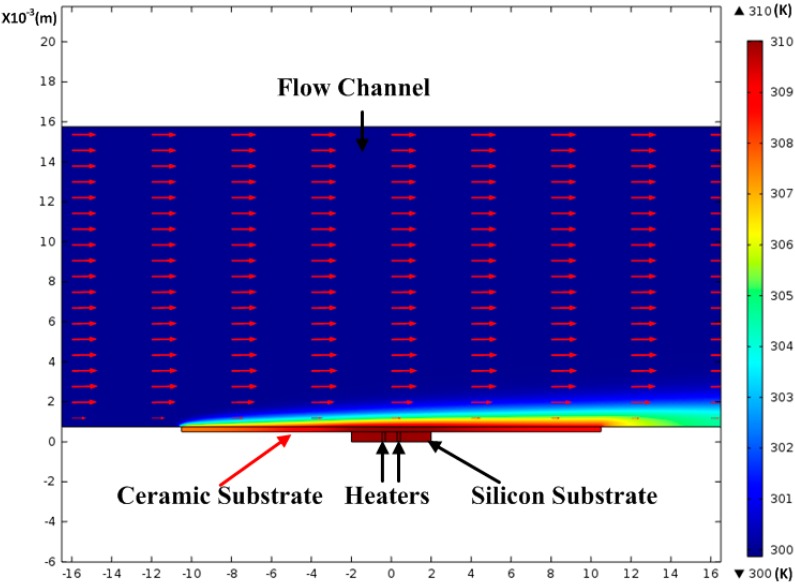
The simulation result of the thermal wind sensor. The ambient temperature is 300 K, and the average temperature of the sensor is 310 K. The wind speed is 2 m/s.

A 2D FEM model of the thermal wind sensor is built with COMSOL Multiphysics 4.4 software to investigate the temperature effects on the performance of the wind sensor. As shown in Figure 3, the sensor model is composed of four parts, including a flow channel, a ceramic substrate, a silicon chip and two heaters. Here, the thermopiles are omitted to shorten the simulation time. The thickness and length of the silicon chip are 0.5 and 4 mm, whereas the thickness and length of ceramic disk are 0.25 and 21 mm. The distance between heaters is 0.6 mm, and the length of the heater is 0.15 mm. At the fluid inlet, the ambient temperature is set to 300 K and the laminar inlet flow rate is 2 m/s. At the flow outlet, the outlet boundary condition is set to outflow and the flow boundary condition is set to zero relative pressure. The top and bottom surfaces of the flow channel are set with slip and no-slip wall conditions, respectively. Besides, the top surface of the flow channel is set to 300 K. The parts of the bottom surface not overlapped with the ceramic disk, the ceramic bottom surface, and the silicon bottom surface are set as thermal insulation. The initial temperatures of the silicon substrate and ceramic disk are set to 300 K. The temperature of the heaters is set to 310 K and the temperature difference between the sensor and the ambient temperature is kept 10 K. The upstream and downstream sensing points are placed symmetrically on the surface of the sensor chip, and the distance of them is 3 mm. Figure 3 shows the simulation results of temperature distribution and flow velocity distribution in the model.

**Figure 4:**
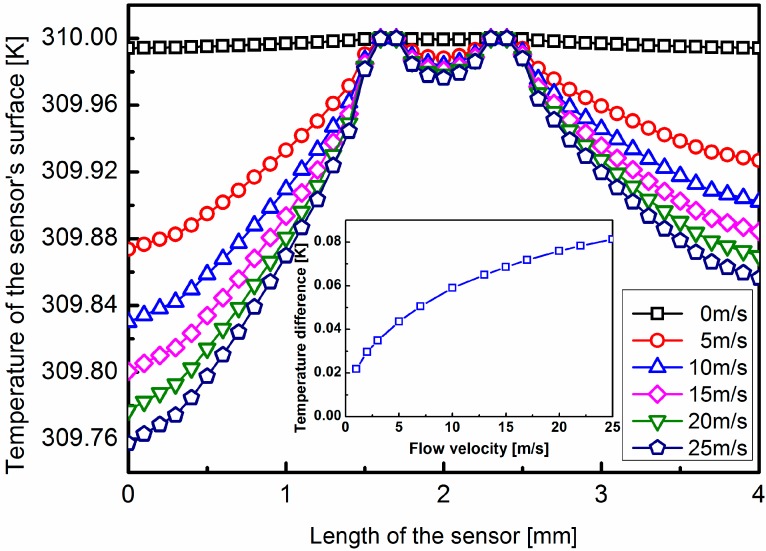
Simulation results of the temperature distribution on the surface of the chip. The wind speed changes from 0 to 25 m/s in a step of 5 m/s. The ambient temperature is 300 K, and the temperature of the heaters is 310 K. The upstream sensing point is at the position of 0.5 mm of the lateral axis, whereas the downstream sensing point is at the position of 3.5 mm of the lateral axis.

Then the average velocities vary from 0 to 25 m/s in steps of 5 m/s. The simulation results are shown in Figure 4. At zero flow, the produced thermal profile is symmetrical with respect to the center point. As the flow velocity increasing, the symmetrical temperature distribution is distorted. The temperature difference between the upstream and downstream sensing points increases with the flow speed.

**Figure 5:**
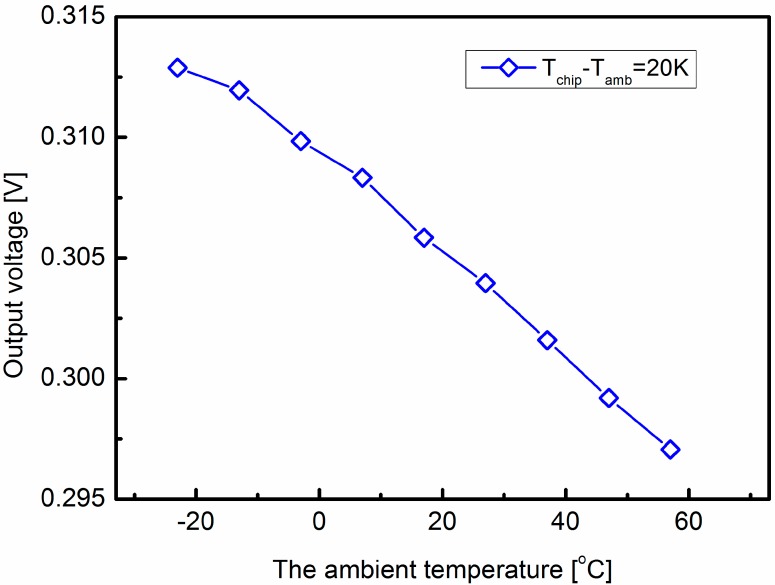
Simulation results of the effect of the thermophysical properties of the airflow on the output voltage at different temperatures.

**Figure 6:**
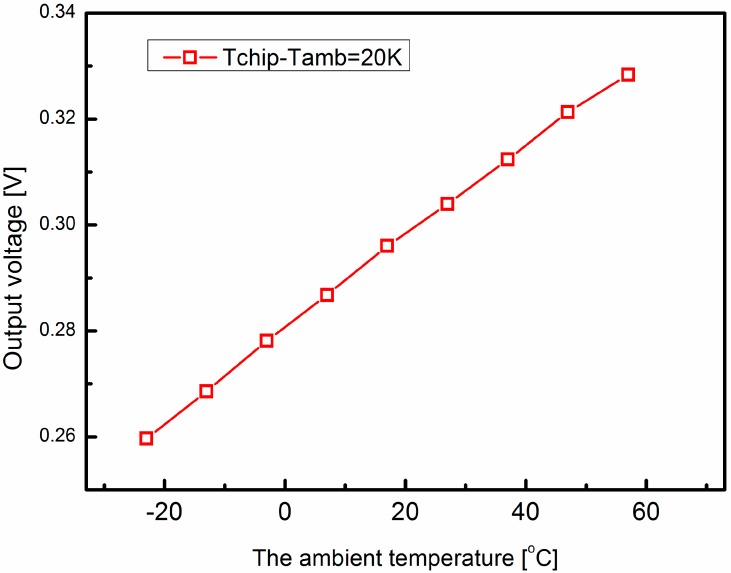
Simulation results of the effect of the silicon and ceramic substrates on the output voltage at different temperatures. The output voltage curve is a linear function of the ambient temperature.

**Figure 7:**
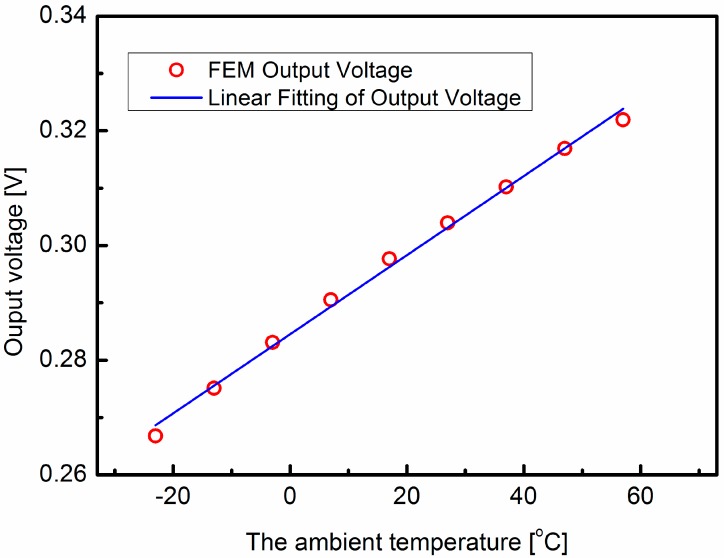
Simulation results of the output voltage of the wind sensor. The output of the wind sensor is a linear function of temperature.

As we reported, the thermal conductivities of the silicon and ceramic substrates are temperature dependent, and the thermophysical properties of the airflow are also functions of the ambient temperature. The data of thermophysical properties of dry air and the thermal conductivity of silicon and ceramic at different temperatures can be found in [23,24]. First of all, the effect of the thermophysical properties of the airflow on the temperature difference on the sensor surface was studied. The material properties of silicon and ceramic are set according to the data sheet for 320 K [23]. At the same time, the thermophysical properties of dry air are set according to different temperatures [24]. The inlet flow rate is 15 m/s, the temperature difference between the sensor and the flow is 20 K. The temperature difference on the sensor surface is converted into the output voltage signal by the thermopiles and amplified 500 times. Each thermopile consists of 10 polysilicon/Al thermocouples with an estimated sensitivity of 2.095 mV/K. Figure 5 shows the simulation results of the output voltages at different temperatures. The temperature difference decreases linearly by less than 5% when the temperature increases 80 °C. Next, the effect of the conductivities of silicon and ceramic substrates are investigated. As shown in Figure 6, the output voltage has a linear relationship with the ambient temperature. When the temperature increases 80 °C, the temperature difference increases 26.4%. It is obvious that the temperature effect of the thermal conductivity of the substrate plays dominant role, which is consistent with our previous research [22]. The output voltage of the thermal wind sensor at different temperatures is shown in Figure 7. This indicates that the output voltage is approximately a linear function of the ambient temperature.

## 4. Experimental Results and Discussion

A fabricated two-dimensional thermal wind sensor consists of four polysilicon heaters, four polysilicon/Al thermopiles, and a central transistor. The sensor chip has been fabricated using a commercial IC fabrication process in a CSMC foundry. Details of the thermal wind sensor can be found in [19]. The size of the sensor chip is 4 mm × 4 mm. The sensor is heated by the four polysilicon resistors, each of which has a resistance of 400 Ω. The four polysilicon/Al thermopiles located symmetrically around the central point measure the temperature difference between the upstream and downstream points on the surface of the sensor chip. Each thermopile is composed of 10 p-doped polysilicon/Al thermocouples. Due to the high impurity concentration (10^26^ m^−3^) the Seebeck coefficient of the thermocouple shows a negligible change with the temperature [22]. Therefore, the sensitivity of the thermopile is nearly constant. The central transistor is connected as a diode which has a temperature coefficient of −1.5 mV/K. The diode measures the average temperature of the sensor, and the chip temperature is been used in the outer control circuit to maintain a constant temperature difference between the sensor and the ambient temperature. The silicon chip is directly mounted on a ceramic substrate which provides a smooth surface. This structure also helps to shield the sensor from any dust in the airflow [1,19].

**Figure 8:**
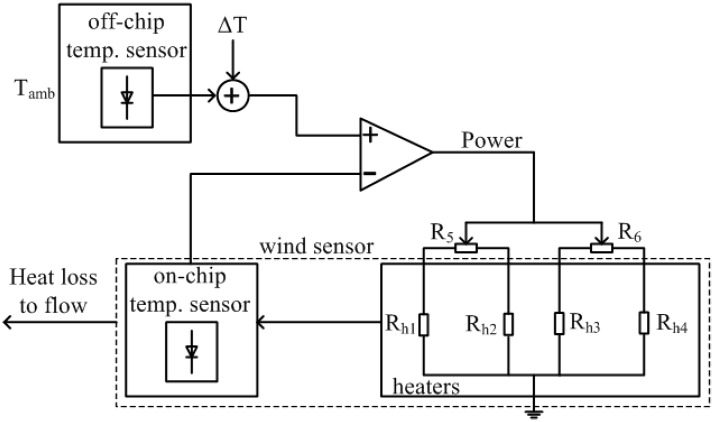
Scheme of the control circuit used to maintain a constant temperature difference between the sensor and the airflow.

The wind sensor is operated in constant temperature difference (CTD) mode which maintains a constant temperature difference between the chip and the airflow. The scheme of the control circuit is shown in Figure 8, where the sensor temperature is measured with the on-chip temperature sensor, and the flow temperature with the off-chip temperature sensor on a similar unheated reference chip. The temperature difference between the sensor and the flow is set as ΔT. For the heating of the sensor, multiple heating resistors have been used, indicated by R_h1_ to R_h4_. By adding variable resistances R_5_ and R_6_ in the heating circuit of each pair of heating resistors, the sensor heating can be balanced in order to remove thermal offset, *i.e.*, the presence of a temperature difference in the absence of flow. The whole power consumption of the wind sensor is 400 mW.

**Figure 9:**
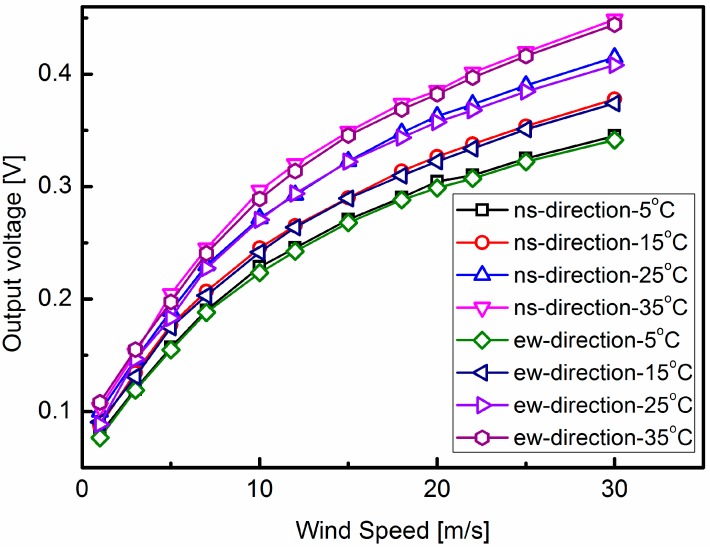
The output voltages in north-south and east-west directions at different ambient temperatures ranging from 5 °C to 35 °C.

**Figure 10:**
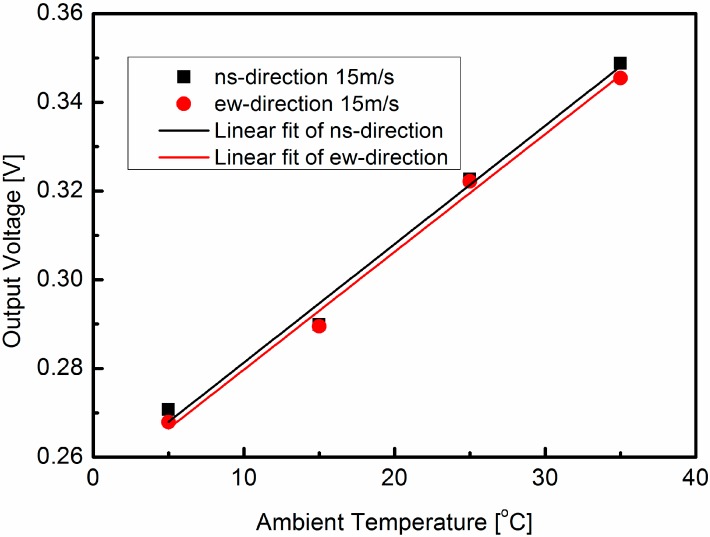
The output voltages in north-west and east-west directions have the ambient temperature when the wind speed is 15 m/s.

Measurements have been performed by installing the sensor in a wind-tunnel with the wind speed up to 40 m/s and a full range of 360°. Figure 9 shows the measurement results of the wind sensor at different temperatures of 5, 15, 25 and 35 °C. It is shown that the output voltages in the north-south direction and east-west direction increase with the ambient temperature, and the output voltage curves in the north-south direction almost coincide with the east-west direction ones at identical temperatures. When the wind speed is 15 m/s, the output signals in the north-south and east-west directions are measured and plotted in Figure 10. Comparing the simulation results in Figure 7 and the measured output in Figure 10, the output voltage curves are seen to be linear functions of temperature. Some factors contribute to the difference between the simulation results and the measured output. For instance, some simplification and approximation have been assumed in the COMSOL simulation, and there are some deviations between the theoretical calculation of Seebeck coefficient and actual measurement.

The output signals in the north-south direction and east-west direction are nearly linear functions of the ambient temperature. From a least-squares fit of the data, the parameters in the north-south and east-west directions can be obtained. *A_ns_* = 6.574 × 10^−2^ V/(m/s)^1/2^, *B_ns_* = 6.894 × 10^−4^ V/(m/s)^1/2^/°C, *A_ew_* = 6.537 × 10^−2^ V/(m/s)^1/2^, *B_ew_* = 6.842 × 10^−4^ V/(m/s)^1/2^/°C. Comparing the values of parameters *A_ns_*, *A_ew_*, *B_ns_* and *B_ew_* with the values of parameters in [13], the huge discrepancy primarily derives from the constant temperature difference between the sensor and the airflow. According to the semi-empirical model [13], the constant temperature difference between the sensor and the airflow is incorporated into these fitting parameters *A* and *B*. Besides, some little deviations in the fabrication and packaging process also contribute to these discrepancies.

**Figure 11:**
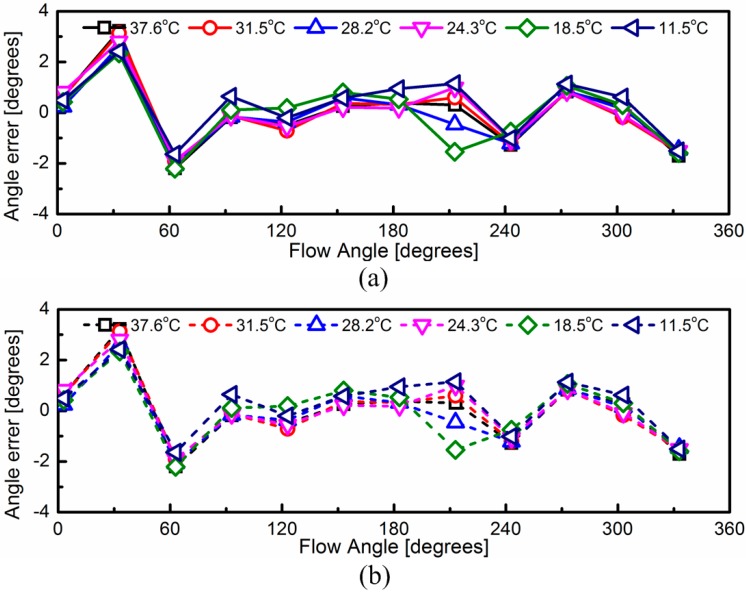
(**a**) The wind speed direction before considering the temperature dependence of the output signals; (**b**) The wind speed direction after considering the temperature dependence of the output signals.

In Figure 11a, the temperature effect on the output signals has not been compensated. The wind direction measurement results are directly calculated by [20]:

(5) $$\phi \text{=}\tan^{-1}\left(\frac{V_{ns}\cdot \cos\left(\varepsilon_{ew}\right)-V_{ew}\cdot \sin\left(\varepsilon_{ns}\right)}{V_{ns}\cdot \sin\left(\varepsilon_{ew}\right)+V_{ew}\cdot \cos\left(\varepsilon_{ns}\right)}\right)$$

On the basis of Equation (4), the temperature effect on the output signals has been compensated in Figure 11b. It can be seen that the measurement results of the wind direction have similar accuracies before and after temperature compensation. This is because that the wind sensor has a symmetrical structure.

When the ambient temperature varies, the ambient temperature effects on the output voltages in these two directions are similar. Furthermore, the parameters *A_ew_*, *B_ew_*, *A_ns_* and *B_ns_* only depend on the thermophysical properties of the airflow, the thermal conductivities of the silicon and ceramic substrates, the Seebeck coefficients of the thermocouples and the temperature difference between the sensor and the airflow. In the same sensor, these physical parameters and the temperature difference between the sensor and the airflow are almost the same in the east-west and north-south directions. This can be proved by the value of the parameters *A_ns_*, *B_ns_*, *A_ew_* and *B_ew_* for the output signal of the wind sensor when the wind speed 15 m/s in Figure 10.

## 5. Conclusions

Solid thermal wind sensors play an important role in the agriculture, industry and meteorology fields. However, the performance of thermal wind sensors is affected by the ambient temperature. In previous works, the effect of the ambient temperature on the wind speed measurement has been studied and a new temperature compensation method has been presented. However, how the ambient temperature affects the wind direction measurement has not been investigated. In this paper, a temperature model for the wind direction measurement is proposed. It is found that the output signals in two orthogonal directions can determine the wind direction, and each output signal is a linear function of the temperature. For the wind sensor with a symmetrical structure, the temperature drift characteristics in the two orthogonal directions are quite similar, therefore, the wind direction of the wind sensor is approximately impendent of the ambient temperature.
